# Application of machine learning algorithms in predicting new onset hypertension: a study based on the China Health and Nutrition Survey

**DOI:** 10.1265/ehpm.24-00270

**Published:** 2025-01-10

**Authors:** Manhui Zhang, Xian Xia, Qiqi Wang, Yue Pan, Guanyi Zhang, Zhigang Wang

**Affiliations:** 1 Department of Disease Control and Prevention, The Seventh Medical Center of Chinese PLA General Hospital, Beijing, China; 2 Office of Epidemiology (Technical Guidance Office for Patriotic Health Work), Chinese Center for Disease Control and Prevention, Beijing, China; 3 Department of Neurology, Beijing Tiantan Hospital, Capital Medical University, Beijing, China; 4 China National Clinical Research Center for Neurological Diseases, Beijing, China

**Keywords:** Machine learning algorithms, Prediction, New onset hypertension, CHNS

## Abstract

**Background:**

Hypertension is a serious chronic disease that can significantly lead to various cardiovascular diseases, affecting vital organs such as the heart, brain, and kidneys. Our goal is to predict the risk of new onset hypertension using machine learning algorithms and identify the characteristics of patients with new onset hypertension.

**Methods:**

We analyzed data from the 2011 China Health and Nutrition Survey cohort of individuals who were not hypertensive at baseline and had follow-up results available for prediction by 2015. We tested and evaluated the performance of four traditional machine learning algorithms commonly used in epidemiological studies: Logistic Regression, Support Vector Machine, XGBoost, LightGBM, and two deep learning algorithms: TabNet and AMFormer model. We modeled using 16 and 29 features, respectively. SHAP values were applied to select key features associated with new onset hypertension.

**Results:**

A total of 4,982 participants were included in the analysis, of whom 1,017 developed hypertension during the 4-year follow-up. Among the 16-feature models, Logistic Regression had the highest AUC of 0.784(0.775∼0.806). In the 29-feature prediction models, AMFormer performed the best with an AUC of 0.802(0.795∼0.820), and also scored the highest in MCC (0.417, 95%CI: 0.400∼0.434) and F1 (0.503, 95%CI: 0.484∼0.505) metrics, demonstrating superior overall performance compared to the other models. Additionally, key features selected based on the AMFormer, such as age, province, waist circumference, urban or rural location, education level, employment status, weight, WHR, and BMI, played significant roles.

**Conclusion:**

We used the AMFormer model for the first time in predicting new onset hypertension and achieved the best results among the six algorithms tested. Key features associated with new onset hypertension can be determined through this algorithm. The practice of machine learning algorithms can further enhance the predictive efficacy of diseases and identify risk factors for diseases.

## 1. Background

Hypertension, a severe chronic condition, significantly contributes to various cardiovascular diseases, affecting vital organs like the heart, brain, and kidneys [1]. It is estimated that 33% of adults aged 30–79 worldwide suffer from hypertension [2], which accounts for over 60% of global disability-adjusted life years (DALYs) attributed to chronic non-communicable diseases. These diseases are the primary cause of death globally, many of which can be preventable through effective control and treatment of hypertension [3, 4]. In 2021, hypertension was the second-leading risk factor globally, responsible for 7.0% of all DALYs and more than ten million deaths annually [1, 5]. The prevalence of hypertension, which increases with age, has surged due to the evolution of modern lifestyles and an aging population, doubling from 650 million in 1990 to 1.3 billion in 2019 [1].

Research indicates a strong correlation between hypertension and factors such as age, Body Mass Index (BMI), smoking, physical activity, cardiovascular diseases, and diabetes [6]. In recent decades, China has experienced a shift in traditional lifestyles due to rapid economic development, with increases in caloric intake and sedentary behaviors likely elevating hypertension rates. Although some lifestyle indicators have improved, they remain below recommended levels [7]. Despite increasing focus by the Chinese government on managing hypertension and other chronic diseases, awareness, treatment, and control of hypertension are still far from optimal [8].

The application of artificial intelligence and machine learning in medical fields has advanced significantly, particularly in predicting diseases and their outcomes. Our study examines various machine learning models to predict hypertension, aiming to facilitate early identification and timely health interventions [9, 10].

Traditional machine learning methods such as Logistic Regression, Support Vector Machines (SVM), and tree ensemble models like XGBoost [11] and LightGBM [12] remain the mainstream choices for tabular data modeling due to their robustness on structured data. However, these methods require extensive manual feature selection operations, and their effectiveness largely depends on the quality of the original features produced by feature engineering in various domains. With the popularity of deep learning, researchers have attempted to introduce end-to-end deep learning modeling to reduce the reliance on feature engineering in processing tabular data [13–15]. However, recent studies have shown that deep learning does not significantly and consistently outperform tree models on tabular data [16]. Recent research [17, 18] indicates that algorithmic feature interactions play a critical role in mitigating the risk of underfitting, which often stems from the lack of essential features, through the identification and extraction of key additive and multiplicative interaction candidates. Moreover, the incorporation of learnable prompt tokens as queries for both addition and multiplication helps to avoid potential overfitting caused by feature redundancy. Arithmetic feature interaction is crucial in deep tabular learning. Therefore, in this study, we introduce an arithmetic feature interaction module and combine it with the Transformer [19] model for deep tabular data modeling.

## 2. Methods

### 2.1 Study design and population

The China Health and Nutrition Survey (CHNS) is a collaborative international project between the Carolina Population Center at the University of North Carolina at Chapel Hill and the National Institute of Nutrition and Health at the Chinese Center for Disease Control and Prevention. This ongoing open cohort study aims to evaluate the impact of health policies and family planning programs initiated by both national and local governments. Additionally, it examines the effects of socio-economic transformations in China on the health and nutritional status of its population. The study analyzes the repercussions of these changes through shifts in community programs and organizations, as well as alterations in economic, demographic, and social factors at the household and individual levels [20, 21]. The survey aims to assess the impact of health, nutrition, and family planning policies and programs implemented by national and local governments, as well as to understand how socio-economic transitions in Chinese society influence the population’s health and nutritional status.

**Fig. 1:**
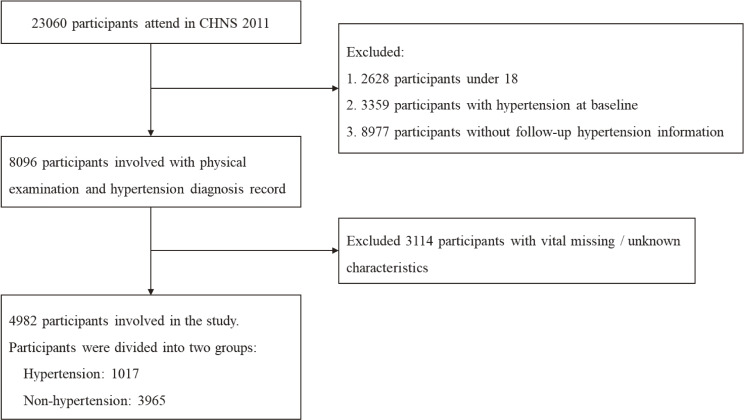
Participants information. The selection process for the study population is described, after data cleaning, a total of 4,982 participants were included in the study.

Due to differences in follow-up intervals and population demographics, we used data from the CHNS cohort from 2011 to 2015 for prediction. The study population was sourced from 12 provinces, autonomous regions, or municipalities, including Beijing, Liaoning, Heilongjiang, Shanghai, Jiangsu, Shandong, Henan, Hubei, Hunan, Guangxi, Guizhou, and Chongqing. The inclusion criteria were (1) age ≥18 years; (2) participants who were not hypertensive in 2011 and had follow-up outcomes available in 2015. The exclusion criteria were (1) age <18 years; (2) participants who missed critical information such as blood pressure measurements, hypertension diagnosis, or metrics such as weight, height, waist, and hip circumference. Finally, a total of 4,982 participants were included. See Fig. 1.

Blood Pressure was measured by trained examiners using a mercury sphygmomanometer at three different consecutive times with 3–5 min intervals on one visit. In our study, the average of three measurements of systolic and diastolic blood pressure was used. All physical examinations were performed at the same location and followed the same protocol at each study visit.

### 2.2 Definition

Hypertension—Adopting the standards of the International Hypertension League, hypertension was defined as a blood pressure ≥140/90 mmHg, a history of hypertension, or using antihypertensive drugs during the previous 2 weeks.

BMI was calculated as weight (kilogram) divided by height (meter) squared.

Waist-to-Hip Ratio (WHR) was calculated as waist circumference (cm) divided by hip circumference (cm).

### 2.3 Data preprocessing

In this study, we utilized 14 categorical features, which include province, urban/rural site, medical insurance status, employment status, diabetes diagnosis, gender, smoking status, drinking status, two representations of educational level (7 levels of none, grad from primary, lower middle school degree, upper middle school degree, technical or vocational degree, university or college degree, master’s degree or higher and 3 levels of lower than high school, high school, higher than high school), marital status, category of BMI, category of total individual income inflated to 2015 (the per capita household income adjusted to the price levels at the end of 2015), and tea consumption habit. Additionally, we analyzed 10 numerical features: daily bedrest duration, age, triceps skinfold thickness (TSF), hip and waist circumferences, WHR, height, weight, BMI, and total individual income inflated to 2015. The initial processing involved samples exhibiting partial anomalies. Specifically, samples displaying waist and hip circumferences outside the 40–180 cm range, waist-to-hip ratios below 0.6 or above 1.3, or BMI values below 10 or exceeding 50 were subject to adjustments. The abnormal feature values in these instances were substituted with values computed via the k-nearest neighbors algorithm (KNN) [22]. This methodology entailed identifying the five closest matching samples and recalculating the abnormal features based on the most akin attributes among these samples before replacing the discrepant values. Subsequently, numerical features were categorized based on predefined thresholds: age into five groups (<25, 25–40, 40–55, 55–65, >65), TSF into four groups (<10, 10–16, 16–22, >22), WHR into five groups (<0.8, 0.8–0.85, 0.85–0.9, 0.9–1, >1), hip circumference into four groups (<80 cm, 80–90 cm, 90–100 cm, >100 cm), and waist circumference into four groups (<70 cm, 70–80 cm, 80–90 cm, >90 cm), creating new categorical features. In summary, our study utilized a total of 29 variables, including 10 numerical features and 19 categorical features. The dataset was then split into training and testing sets in a 9:1 ratio, using a random seed of 3123 for replication purposes in model training and evaluation.

### 2.4 Statistical analysis

A descriptive analysis of baseline characteristics was performed using the mean ± standard deviation (SD) or median (interquartile range) for continuous variables and frequencies (percentages) for categorical variables. The Wilcoxon non-parametric test and the chi-squared test were employed to compare differences between groups. A statistically significant value was considered when *P* < 0.05 on both sides. Statistical analysis was performed using SAS 9.4. The development environment for this experiment included Python 3.8.18 and PyTorch 2.2.0. Training was performed on a GeForce RTX A6000 with a batch size of 128 for all models.

### 2.5 Algorithm

Machine learning methods have already made significant advancements in numerous fields [23–26]. We selected traditional machine learning algorithms such as Logistic Regression [27], Support Vector Machine [28], XGBoost [11], and LightGBM [12], as well as the deep learning algorithm TabNet, as comparison models for the AMFormer model with the arithmetic feature interaction module, among which XGBoost and LightGBM are tree ensemble models. The following is an introduction to the principles of each model:

#### 2.5.1 Logistic regression

Logistic Regression is a widely used linear classification algorithm that is suitable for binary classification problems. It restricts the model’s output between 0 and 1 by applying a logistic function (i.e., the Sigmoid function), making the output interpretable as a probability. Although simple, Logistic Regression is remarkably effective when dealing with linearly separable data and is widely used in fields such as medicine and finance.

#### 2.5.2 Support vector machines

SVM is a powerful classification method designed to find the optimal boundary between different categories. By maximizing the distance between category boundaries, SVM ensures the model has good generalization ability. SVM can handle linearly inseparable data by introducing the kernel trick, making it an effective tool for processing high-dimensional data.

#### 2.5.3 XGBoost

XGBoost is an efficient tree boosting algorithm that uses a gradient boosting framework. It optimizes traditional gradient boosting algorithms by employing techniques such as parallel processing and tree pruning, enhancing training speed and model performance. XGBoost is extensively used in various machine learning competitions, especially exhibiting exceptional performance on structured data.

#### 2.5.4 LightGBM

LightGBM is a gradient boosting learning algorithm that trains and predicts faster than traditional gradient boosting methods by utilizing histogram-based decision tree algorithms. It also employs a leaf-wise growth strategy with depth limitation, effectively reducing overfitting and delivering better performance on large datasets.

#### 2.5.5 TabNet

TabNet is a deep learning model based on the attention mechanism, designed specifically for tabular data. By utilizing a sparse attention mechanism, TabNet selectively focuses on subsets of input features, thereby enhancing the model’s interpretability and accuracy. This approach showcases superior performance in handling complex non-linear relationships, especially on structured data.

#### 2.5.6 AMFormer

**Fig. 2:**
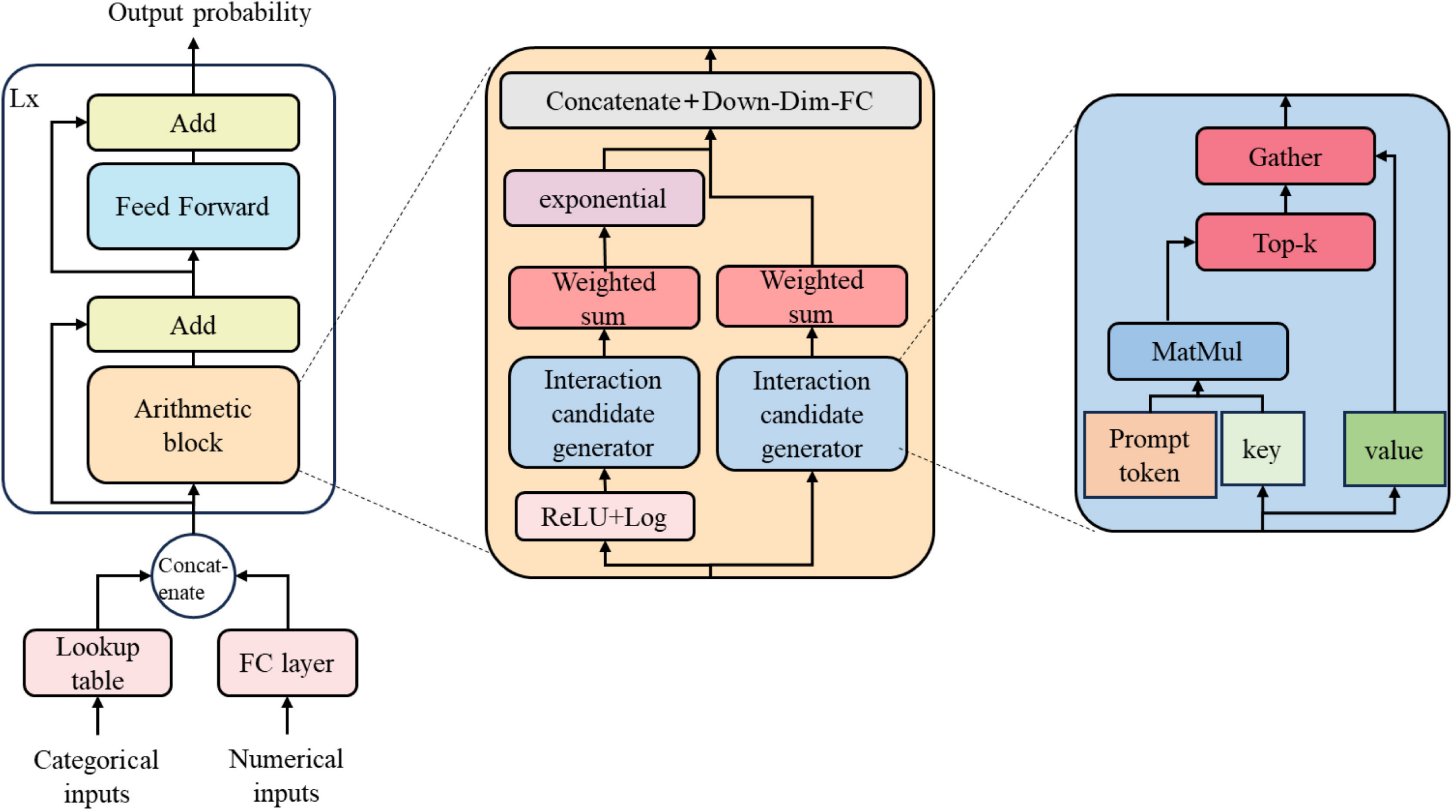
Overview of the AMFormer workflow.

As shown in Fig. 2, the AMFormer architecture draws on the fundamental principles of the classic Transformer framework and significantly enhances the model’s ability to process arithmetic feature interactions by integrating the Arithmetic Block. This architecture initially maps numerical features (such as age and BMI) through a single-input, multiple-output linear layer to form embeddings with strong representational power; for categorical features (such as gender and diabetes diagnosis), it uses multidimensional embedding lookup tables for processing. Subsequently, these initial embeddings are fed into L sequential processing layers, which further enhance feature expression by strengthening the contextual links and element interactions between embeddings. Each layer includes an arithmetic module that enables the interaction between arithmetic features by implementing a parallel attention mechanism for addition and multiplication, thereby effectively improving the model’s processing capability. Additionally, the residual connections and forward propagation networks from the original Transformer structure not only facilitate gradient flow but also optimize feature representation. Based on these enhanced embeddings, AMFormer is capable of outputting the final processing results through a classification or regression head, with a classification head mainly used for model output in this research.

The Arithmetic Block, as the core component of AMFormer, leverages parallel attention mechanisms and prompt tokens to enhance the interaction between arithmetic features. This parallel mechanism processes the embeddings of input features through two independent attention paths in parallel, one responsible for calculating addition interactions and the other for simulating multiplication interactions. It then merges these two types of interaction candidates and integrates them through a fully connected layer, thereby representing the arithmetic combination of input features. Specifically, for the input feature embedding matrix X, in the addition path, embeddings for query, key, and value are generated through trainable parameters and their affinity calculated to generate candidates for the addition interaction. To address the sparsity of interactions, a hard attention mechanism is employed, keeping only the top k affinity entries. The multiplication path processes multiplication interactions on a logarithmic scale before outputting the result. AMFormer merges the outputs of these two paths and promotes complete arithmetic feature interaction through a fully connected layer.

### 2.6 Experiments

#### 2.6.1 Implementation details

In this study, we configured and compared the performance of five contrast models alongside AMFormer. We used grid search [29] for each model to determine the optimal hyperparameters. Specifically, for Logistic Regression (We used Regularized Logistic Regression through the scikit-learn library) and SVM, a grid search was conducted for the penalty parameter C ranging from 0.001 to 100 to find the optimal hyperparameter; for XGBoost and LightGBM, we performed grid searches on maximum depth (2–6) and the number of estimators (50, 100, 150, 200).

Detailing specific parameter settings as outlined: The SVM was implemented using a polynomial kernel function with the penalty parameter C set to 1.0. The XGBoost classifier configuration included using 50 tree estimators for the model with 16 features, with a learning rate of 0.05, a maximum depth of 3, and both subsample and feature sampling rates of 0.8, targeting a binary Logistic Regression; for the model with 29 features, 100 tree estimators and a maximum depth of 4 were used. For the Logistic Regression model, the penalty parameter C was set to 0.01, with the maximum number of iterations adjusted to 1000 to ensure convergence. The LightGBM classifier had similar parameter configurations to XGBoost, including 100 estimators, a learning rate of 0.05, a maximum depth of 4, subsample and feature sampling rates of 0.8, and a target set for binary classification. The TabNet classifier utilizes the Adam optimizer with a learning rate of 0.001 and a learning rate scheduler set at a step size of every 10 epochs and a decay factor of 0.9, selecting “sparsemax” as the mask type.

The AMFormer’s hyperparameter setup includes an embedding vector dimension of 336, model depth of three attention blocks, twelve heads in multi-head attention, dropout rates of 0.5 and 0.4 for the attention and feedforward networks, respectively, feature numbers in subsequent layers set as 200, 100, and 50 with the top k for summation and multiplication within each layer’s groups set at specific values, and the Sigmoid function employed in the output layer.

#### 2.6.2 Evaluation details

In the specific implementation process, we first followed the traditional Logistic Regression prediction approach to select features that might influence the prediction of new onset hypertension. We manually screened variables to form a 16-feature prediction model (including urban/rural site, medical insurance status, employment status, diabetes diagnosis, gender, smoking status, drinking status, 3 levels of educational attainment, marital status, category of BMI, category of total individual income inflated to 2015, tea consumption habit, daily bedrest duration, age, TSF, WHR) to avoid issues like multicollinearity and overfitting in the model. Data mining and machine learning methods can utilize all available variables in the dataset without pre-setting the association between variables or any specific variable’s predictive power. These methods allow the identification of potentially high predictive variables that might be overlooked using more traditional methods such as Logistic Regression [30]. Therefore, based on the 16 features, we constructed additional feature variables, resulting in a prediction model covering 29 features. Considering the employment of machine learning for prognostications, our model could assimilate more variables, thereby drawing out more information. Consequently, we discretized numeric attributes from these manually elected 16 features, thereby cultivating new categorical attributes. The objective was to counter the disruptive implications from statistical outlier in numeric data and apprehend potential nonlinear liaisons in the model more conveniently through discretization. Without affixing supplementary investigation indicators, we augmented the utilization of existing information, thus enhancing the prognosticative effect.

Specifically, numerical features were categorized based on predefined thresholds: age into five groups (<25, 25–40, 40–55, 55–65, >65), TSF into four groups (<10, 10–16, 16–22, >22), WHR into five groups (<0.8, 0.8–0.85, 0.85–0.9, 0.9–1, >1), hip circumference into four groups (<80 cm, 80–90 cm, 90–100 cm, >100 cm), and waist circumference into four groups (<70 cm, 70–80 cm, 80–90 cm, >90 cm), creating new categorical features, thereby formulating 29 features in total.

In our evaluation, we used metrics such as Accuracy (ACC), Matthews Correlation Coefficient (MCC), F1-Score (F1), Area Under the Receiver Operating Characteristic Curve (AUC), Precision, and Recall to comprehensively evaluate the model’s performance. AUC assesses the model’s ability to correctly predict samples. ACC represents the proportion of correctly classified samples. The F1-score represents the harmonic mean of recall and precision. MCC takes into account true positives, true negatives, false positives, and false negatives, making it suitable for imbalanced datasets. Deep learning models output probabilities rather than specific categories, usually requiring a threshold to determine whether the model’s prediction is a positive or negative sample. Unlike traditional methods that manually select a threshold, we followed a setup from previous studies [32], where the model outputs the probability for each sample in the test set, and we determined the model classification threshold by maximizing the MCC metric. This threshold is then used to calculate metrics such as ACC, F1, Precision, and Recall. We used a 95% confidence interval for all metrics. To calculate the 95% confidence interval for all metrics, we referred to previous research [32] and used a non-parametric bootstrap method to generate confidence intervals: random samples of size n (equal to the size of the original dataset) were repeatedly resampled 100 times with replacement from the original dataset. Additionally, we also present the calibration plots of all models [31].

### 2.7 SHAP interpretability methods

In the field of interpretability of machine learning models, SHAP (SHapley Additive exPlanations) is a widely used method. SHAP draws on the concept of Shapley values from cooperative game theory to quantify the contribution of each feature to the prediction outcome. By analyzing feature combinations across various comparison sets, SHAP computes the marginal contribution of each feature when it participates individually in the model, thus providing a globally consistent measure of feature importance. The SHAP computation process includes three steps:

1. Sampling feature subsets: For a given prediction, determine the marginal gain of adding a particular feature to the existing combination of features. This requires evaluating all feature subsets that include and exclude the feature in question.
2. Marginal contribution calculation: For each feature, compute its average marginal contribution across different feature combinations. Specifically, assuming there are N features, for feature i, calculate its contribution on all possible subsets of size k.
3. Weighted averaging: Using the Shapley value formula, perform a weighted summation of these marginal contributions to derive the SHAP value of feature i. The formula for this value is defined as follows:
$$\left[\phi_{i}=\sum_{S\subseteq F\setminus i}\frac{|S|!(|F|-|S|-1)!}{|F|!}[f(S\cup i)-f(S)]\right]$$
  where, (ϕ*_i_*) represents the Shapley value of the feature (*i*), (*S*) is a subset of features, (*F*) is the set of all features, and (*f*(*S*)) denotes the model output for a subset (*S*).

## 3. Results

### 3.1 Baseline and univariate analysis

The study included 4982 subjects with a mean age of 48.32 ± 13.01 years. During the 4-year follow-up, 1017 participants developed hypertension, of whom 536 (52.70%) were males. Subjects were divided into two groups according to whether they developed hypertension or not, and the distribution of their characteristics is described in Table 1.

**Table 1:** Baseline characteristics of participants stratified by the new onset hypertension.

**Characteristic**	**Total population**	**Non-hypertension**	**Hypertension**	***P* value**
Age, years	48.32 ± 13.01	46.79 ± 12.87	54.29 ± 11.78	<0.001
Gender, male, n (%)	2336(46.89)	1800(45.40)	536(52.70)	<0.001
Rural site, n (%)	3143(63.09)	2433(61.36)	710(69.81)	<0.001
Educational level, n (%)				<0.001
Lower than high school	3206(64.35)	2466(62.19)	740(72.76)	
High school	671(13.47)	544(13.72)	127(12.49)	
Higher than high school	1105(22.18)	955(24.09)	150(14.75)	
Marital status, n (%)				<0.001
Never married	187(3.75)	177(4.46)	10(0.98)	
Married	4510(90.53)	3577(90.21)	933(91.74)	
Divorced	72(1.45)	58(1.46)	14(1.38)	
Widowed	186(3.73)	130(3.28)	56(5.51)	
Separated	27(0.54)	23(0.58)	4(0.39)	
Smoking status, n (%)				0.347
Never	3440(69.05)	2754(69.46)	686(67.45)	
Past	182(3.65)	139(3.51)	43(4.23)	
Present	1360(27.30)	1072(27.04)	288(28.32)	
Drinking status, n (%)				<0.001
Never	3162(63.47)	2521(63.58)	641(63.03)	
Almost every day	497(9.98)	365(9.21)	132(12.98)	
Every week	638(12.81)	504(12.71)	134(13.18)	
≤2 times/month	685(13.75)	575(14.50)	110(10.82)	
Drinking tea, n (%)	2023(40.61)	1635(41.24)	388(38.15)	0.074
Diabetes, n (%)	105(2.11)	76(1.92)	29(2.85)	0.064
Medical insurance, n (%)	4840(97.15)	3847(97.02)	993(97.64)	0.292
Presently working, n (%)	3772(75.71)	3110(78.44)	662(65.09)	<0.001
Time in bed per day, hours	7.76 ± 1.14	7.77 ± 1.12	7.70 ± 1.19	0.048
Triceps skin fold, cm	17.27 ± 7.82	17.13 ± 7.82	17.82 ± 7.81	0.011
Waist-to-Hip Ratio	0.88 ± 0.11	0.87 ± 0.11	0.90 ± 0.12	<0.001
Total Individual income inflated to 2015, yuan				<0.001
<10000	1159(23.26)	868(21.89)	291(28.61)	
[10000, 20000)	1457(29.25)	1164(29.36)	293(28.81)	
[20000, 30000)	1084(21.76)	869(21.92)	215(21.14)	
≥30000	1282(25.73)	1064(26.83)	218(21.44)	
BMI, kg/m^2^				<0.001
<18.5	261(5.24)	222(5.60)	39(3.83)	
[18.5, 24)	2774(55.68)	2313(58.34)	461(45.33)	
[24, 28)	1513(30.37)	1121(28.27)	392(38.54)	
≥28	434(8.71)	309(7.79)	125(12.29)	

Outliers: In the study, 12 individuals (0.24%) with waist circumferences outside the range of 40 to 180 cm, 13 individuals (0.26%) with hip circumferences outside the same range, 28 occurrences (0.56%) where the waist-to-hip ratio (WHR) was below 0.6 or above 1.3, and 20 individuals (0.40%) with BMI values below 10 or above 50 were identified as outliers. Due to some participants exhibiting outliers across different metrics simultaneously, a total of 53 individuals (1.06%) were classified as outliers and were excluded from further model-fitting analysis.

The hypertension group was older, and had higher TSF and WHR compared with the non-hypertension group (*P* < 0.05). The non-hypertension group was more likely to be males, rural residents, current and former smokers, frequent drinkers, unemployed individuals, and had lower education levels, less sleep, lower income, and higher BMI.

### 3.2 Model comparison

Logistic Regression, XGBoost, LightGBM, SVM, and TabNet were our selected models for comparison. As illustrated in Table 2, the Logistic Regression model demonstrated the highest performance in the 16-feature version, achieving an AUC of 0.784(0.775∼0.806).

**Table 2:** Comparison of MCC, F1-score, ACC, Precision, Recall, and AUC results (16 features).

**Model**	**MCC**	**F1-score**	**ACC**	**Precision**	**Recall**	**AUC**
XGBoost	0.370(0.342–0.397)	0.499(0.476–0.513)	0.747(0.716–0.767)	0.375(0.366–0.393)	0.770(0.742–0.785)	0.780(0.7773–0.788)
Logistic Regression	**0.396(0.3376–0.406)**	**0.513(0.496–0.514)**	0.756(0.754–0.771)	0.399(0.387–0.400)	0.714(0.690–0.721)	**0.784(0.775–0.806)**
LightGBM	0.390(0.383–0.413)	0.508(0.493–0.519)	0.751(0.749–0.756)	0.389(0.374–0.395)	0.726(0.719–0.758)	0.782(0.772–0.803)
TabNet	0.339(0.271–0.362)	0.468(0.408–0.471)	**0.784(0.774–0.820)**	**0.421(0.375–0.465)**	0.476(0.448–0.530)	0.736(0.723–0.761)
Support Vector Machine	0.220(0.173–0.236)	0.383(0.340–0.388)	0.719(0.717–0.721)	0.308(0.288–0.316)	0.490(0.414–0.524)	0.680(0.666–0.702)
AMFormer	0.378(0.358–0.388)	0.457(0.456–0.466)	0.609(0.597–0.619)	0.302(0.301–0.306)	**0.952(0.942–0.977)**	0.760(0.753–0.775)

To further optimize prediction efficiency, we developed a predictive model utilizing 29 features. As illustrated in Table 3, the AMFormer model demonstrated the highest efficacy, achieving an AUC of 0.802(0.795∼0.820). Logistic Regression followed closely with an AUC of 0.788(0.776∼0.811), marking it as the second most effective model. The tree ensemble algorithms showed varied performance; specifically, XGBoost attained an AUC of 0.748(0.735∼0.773), while LightGBM reached an AUC of 0.753(0.741∼0.775). Moreover, the SVM yielded an AUC of 0.702(0.689∼0.726), whereas the deep learning architecture TabNet recorded an AUC of 0.734(0.722∼0.755).

**Table 3:** Comparison of MCC, F1-score, ACC, Precision, Recall, and AUC results (29 features).

**Model**	**MCC**	**F1-score**	**ACC**	**Precision**	**Recall**	**AUC**
XGBoost	0.300(0.248–0.328)	0.441(0.398–0.463)	0.712(0.697–0.725)	0.341(0.299–0.349)	0.621(0.595–0.693)	0.748(0.735–0.773)
Logistic Regression	0.400(0.384–0.403)	0.495(0.470–0.508)	0.765(0.764–0.767)	0.374(0.363–0.393)	0.710(0.678–0.714)	0.788(0.776–0.811)
LightGBM	0.347(0.283–0.360)	0.474(0.421–0.488)	**0.768(0.757–0.778)**	**0.388(0.352–0.404)**	0.575(0.524–0.617)	0.753(0.741–0.775)
TabNet	0.308(0.292–0.337)	0.451(0.434–0.462)	0.717(0.713–0.743)	0.347(0.331–0.357)	0.647(0.632–0.655)	0.734(0.722–0.755)
Support Vector Machine	0.235(0.206–0.256)	0.400(0.376–0.401)	0.647(0.641–0.653)	0.283(0.272–0.286)	0.667(0.609–0.691)	0.702(0.689–0.726)
AMFormer	**0.417(0.400–0.434)**	**0.503(0.484–0.505)**	0.670(0.669–0.675)	0.341(0.330–0.345)	**0.930(0.905–0.966)**	**0.802(0.795–0.820)**

Further analysis from Table 2 revealed the comparative results of AMFormer with the five models in terms of MCC, F1, ACC, Precision, and Recall. AMFormer exceled in both MCC and F1, which were critical indicators of comprehensive model efficacy, achieving an MCC of 0.417(0.400∼0.434) and an F1 score of 0.503(0.484∼0.505). This proved that AMFormer was superior to other comparative models. Our model demonstrated a recall rate of 0.930(0.905∼0.966), which was, on average, 30.16% superior to the five other models assessed. This suggested that our model exhibited a high sensitivity towards positive samples, resulting in a significant reduction in missed diagnoses, particularly relevant in the application of AI for extensive disease screening. The results obtained from the comparative models indicated that tree ensemble models and Logistic Regression continued to excel over alternative models in tabular data modeling. By effectively managing categorical and numerical data separately and integrating an arithmetic feature interaction module, AMFormer achieved accurate modeling of complex data patterns, thereby outperforming traditional machine learning models.

We also report calibration plots for all models [31]. As shown in Fig. 4, XGBoost, Logistic Regression, LightGBM, TabNet, and AmFormer all exhibit good calibration. The probabilities predicted by XGBoost, LightGBM, and TabNet are slightly underestimated, while in the case of Logistic Regression, the model tends to underestimate the probability of events occurring when the predicted probabilities are low. In contrast, the AMFormer model generally has better predictive performance overall.

## 4. Discussion

Our study provides a method for identifying risk factors for new onset hypertension and predicting them using machine learning algorithms. Among these six algorithms, AMFormer exhibited the best performance. These results indicate that machine learning can effectively predict the risk of new onset hypertension in public health practices. Visualization of feature importance provides insights into key features and their associated risks. Moreover, our analysis revealed an interesting phenomenon. In the process of building models through manual feature selection, the traditional Logistic Regression method achieved optimal results. After processing the data to allow more features into the model, the machine learning algorithms further improved the prediction efficiency of the model. Compared to traditional statistical methods like linear regression, machine learning algorithms offer greater flexibility and scalability, enabling elevated efficiency in predictions [33]. Additionally, traditional prediction models using carefully selected and assessed features also yield good results. Complex machine learning models such as XGBoost, LightGBM, and deep learning models often have greater expressive power, but at the same time, they are more prone to overfitting, especially when there are fewer features. The simplicity of Logistic Regression gives it a certain robustness, allowing it to avoid overfitting and perform well.

To analyze the contributions of 10 numerical features and 19 categorical features (including 5 newly constructed features) to our prediction task, as well as the interpretability of the model, we employed SHAP values [34] for the analysis of feature contributions. SHAP values, based on game theory, are used to explain the predictions of machine learning models. By quantifying the contribution of each feature to the model’s prediction outcome, SHAP values offer a way to understand the decision-making process of the model. This method not only identifies the features that significantly impact the model’s predictions but also elucidates the manner in which these features affect individual outcomes. The application of SHAP values enhances the transparency and interpretability of the model, making it exceptionally appropriate for scenarios that require detailed explanations of model decisions. In our study, we used the model-agnostic KernelExplainer and randomly sampled 100 out of 499 samples from the test set to analyze the SHAP values between features and prediction outcomes.

**Fig. 3:**
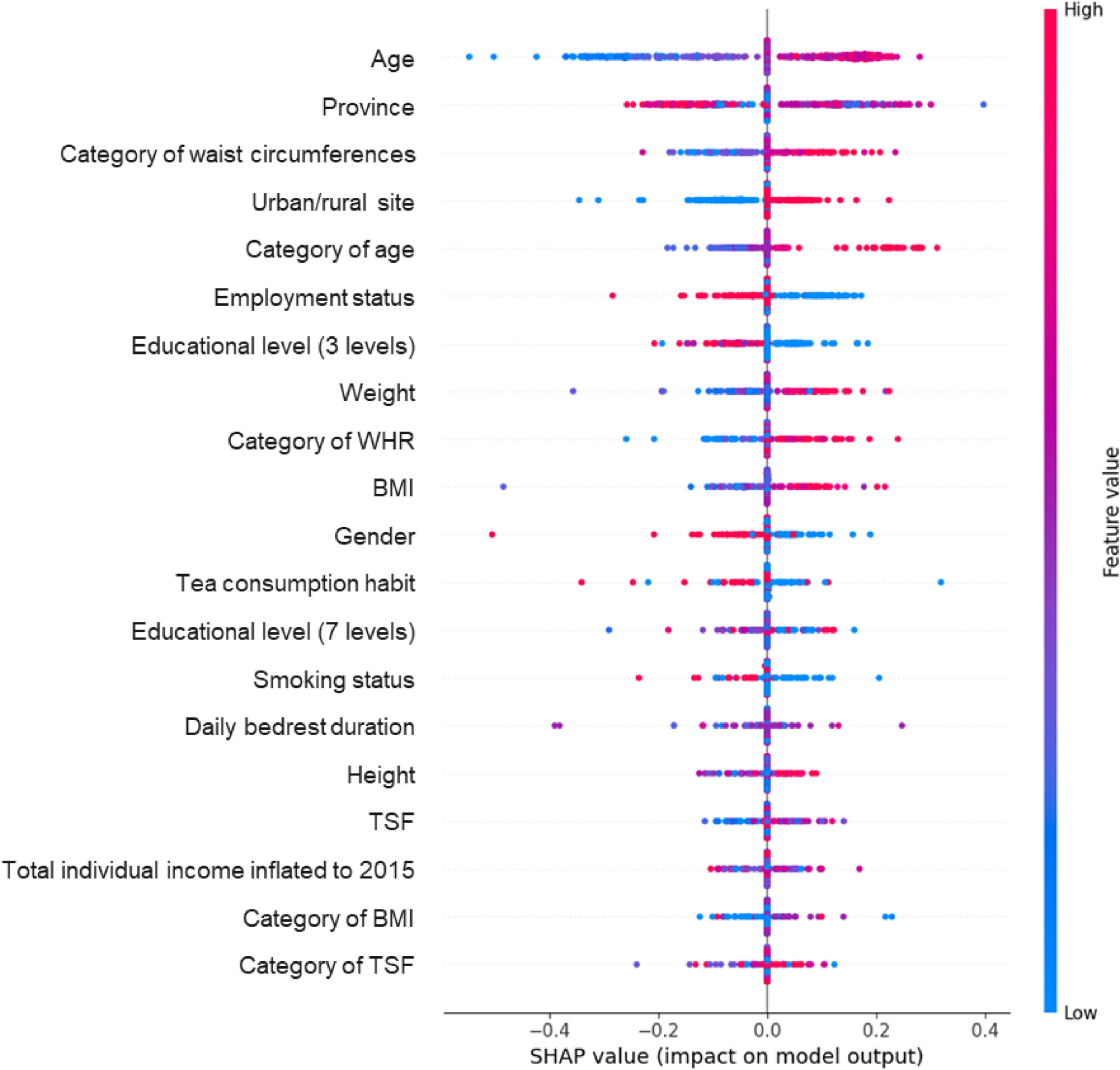
SHAP values in the AMFormer model. It displays the prediction importance of the AMFormer 29-feature model, highlighting the SHAP values of the top 20 most important features.

The Fig. 3 highlights the top 20 features critical to the predictive analysis undertaken in this research. Each point in the diagram represents a sample; the color indicates the magnitude of each feature’s value, with redder indicating a larger value and bluer indicating a smaller value (for example, the older the age, the redder the color; the younger the age, the bluer the color); the vertical axis represents features, sorted by feature importance from top to bottom. The horizontal axis is the SHAP value calculated from the formula, with positive SHAP values indicating that the feature value causes the prediction value to increase (i.e., makes the prediction result more towards 1), and negative SHAP values indicating that it causes the prediction value to decrease (i.e., makes the prediction result more towards 0). It can be seen that the most important feature for this prediction task is age, with increased age correlating with a higher likelihood of positive predictions; it is followed by a certain relationship with region, for example, province and whether it is rural or urban; in addition, there is also a relation with waist circumference, where a larger waist circumference is more likely to be predicted as positive; similar patterns are observed with BMI values and WHR. Furthermore, there is an inverse relationship with employment status and level of education, with unemployed individuals and those with lower education levels being more likely to be diagnosed as positive.

**Fig. 4:**
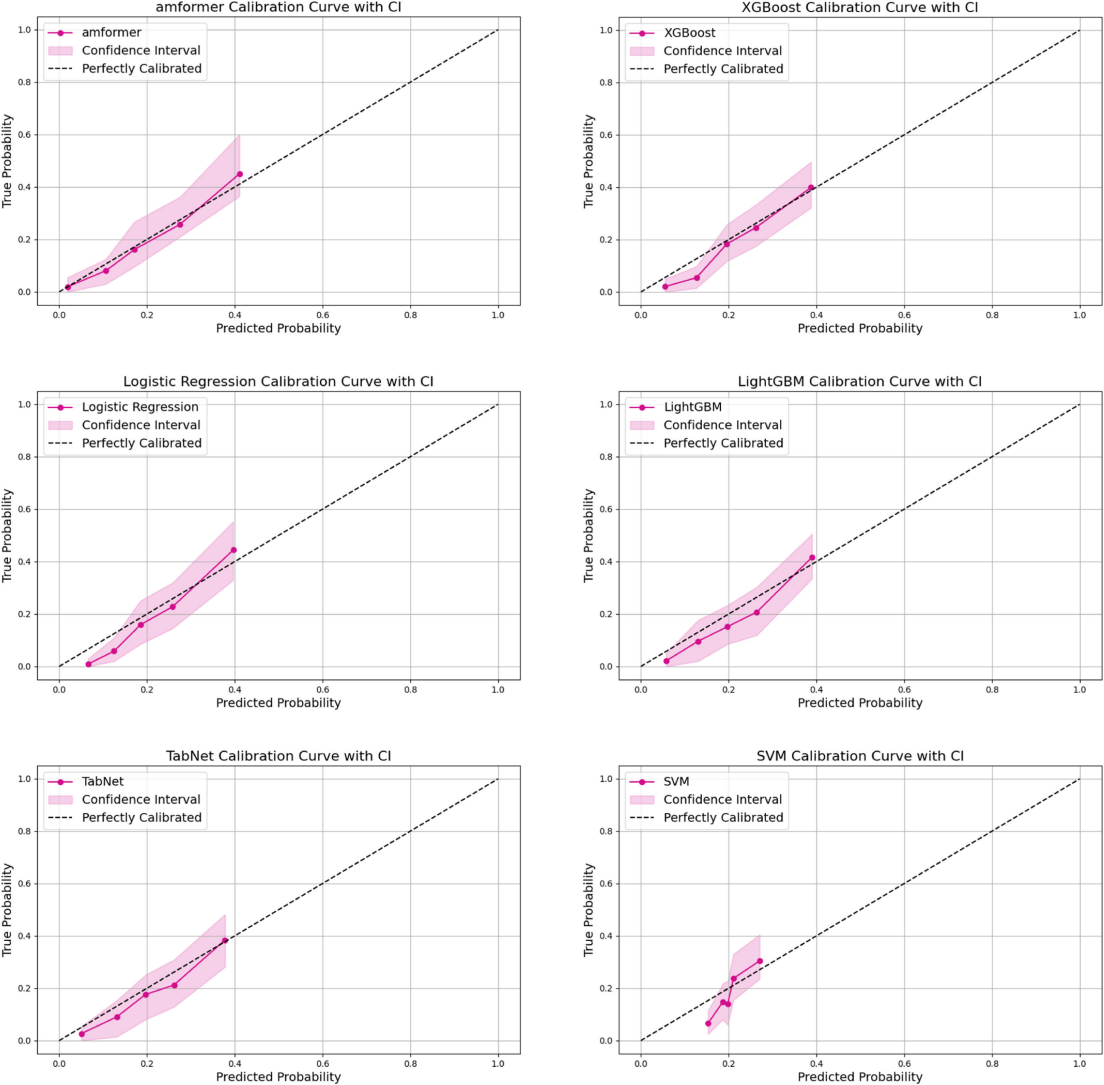
Calibration plots of six 29-feature models.

Our research indicates that age is the most important feature. With increasing age, both blood pressure and the prevalence of hypertension rise [2]. Due to demographic expansion and shifts in age distribution, the global adult population, particularly the elderly, is increasing, leading to a rising prevalence of hypertension and a growing public health challenge. Furthermore, the incidence of hypertension has certain regional factors; higher prevalence rates are found in people from rural areas and the eastern regions [8]. Several studies have shown a clear association between increased blood pressure and weight gain. Data from NHANES indicate that the prevalence of hypertension in obese individuals with a BMI < 30 kg/m^2^ is 42.5%, while it is 15.3% in lean individuals [35]. Similarly, some characteristics associated with obesity, such as hip circumference, weight, waist-to-hip ratio, BMI, and TSF, play a significant role in risk prediction. Hip circumference, ranking third in terms of feature importance in our prediction model, anatomically not only represents fat distribution but also reflects changes in gluteal muscles, skeletal structure (pelvic width), and subcutaneous gluteal fat [36], which may be influenced by lifestyle-related factors such as alcohol consumption, smoking, and physical activity [37]. Hypertension frequently coexists with alterations in lifestyle; in our current research design, we focus on lifestyle factors within the study population, such as smoking and drinking habits, sleep duration, and physical activity. These modifiable risk factors play a significant role in our predictive model and are essential for the prevention of hypertension. Consistent with our findings, short sleep duration is associated with an increased risk of hypertension [38]. In contrast, meta-analyses have found that both short and long sleep durations are associated with increased hypertension risk [39, 40]. The variability in conclusions indicates that sleep duration does not fully reflect sleep patterns, necessitating further research to explore the relationship between the two.

Machine learning is a subfield of artificial intelligence that involves a systematic process of learning from data, training, and accurately predicting future events [41]. We used the AMFormer model, which has shown significantly better performance in fine-grained tabular data modeling, training sample efficiency, and generalization capabilities on synthetic datasets [17]. For the first time, we applied it to hypertension prediction and achieved better predictive efficiency compared to other models in practice, which will help us achieve more accurate results in future disease risk predictions.

There are also several limitations in this study. Firstly, despite our comprehensive feature engineering efforts, the current feature set may still not encompass all potentially important features. Some features that were not included might be crucial for the model’s predictive performance. Furthermore, the choices we made during feature engineering might be biased towards specific datasets, thus failing to accurately reflect the characteristics of a broader population. Secondly, although our model performed well in tests on specific datasets, it has not yet been validated on external datasets. Thirdly, due to limitations in data sources and follow-up duration, we were unable to include additional clinical test data, which resulted in a relatively short prediction timeframe. Therefore, future research should prioritize broader validation of the model among different populations and environments to improve its universality and generalizability. We suggest that future work should expand the feature set and conduct cross-dataset validation to enhance the robustness and practicality of the model.

## 5. Conclusion

We employed the AMFormer model for the first time to predict newly onset hypertension, achieving the highest performance among six tested algorithms. This approach effectively identifies key features associated with the development of hypertension. The implementation of machine learning algorithms can further enhance disease prediction and facilitate the identification of relevant risk factors.

## Abbreviations

CHNS: China Health and Nutrition Survey
DALYs: disability-adjusted life years
BMI: Body Mass Index
SVM: Support Vector Machines
NINH: National Institute of Nutrition and Health
CCDC: Chinese Center for Disease Control and Prevention
WHR: Waist-to-Hip Ratio
TSF: triceps skinfold thickness
KNN: k-nearest neighbors algorithm
SD: standard deviation
SHAP: Shapley additive explanations

## Declarations

### Availability of data and materials

The CHNS datasets can be downloaded at the CHNS home page (https://www.cpc.unc.edu/projects/china).
